# Prevalence and characteristics of mitral valve prolapse in military young adults in Taiwan of the CHIEF Heart Study

**DOI:** 10.1038/s41598-021-81648-z

**Published:** 2021-02-01

**Authors:** Pang-Yen Liu, Kun-Zhe Tsai, Yen-Po Lin, Chin-Sheng Lin, Huan-Chang Zeng, Eiki Takimoto, Gen-Min Lin

**Affiliations:** 1 Division of Cardiology, Department of Internal Medicine, Tri-Service General Hospital and National Defense Medical Center, Taipei, Taiwan; 2 Department of Internal Medicine, Hualien Armed Forces General Hospital, No. 100, Jinfeng St., Hualien City, 970 Taiwan; 3 Department of Critical Care Medicine, Taipei Tzu Chi General Hospital, New Taipei City, Taiwan; 4 GENEUS Medical Technology Co., New Taipei City, Taiwan; 5 Department of Cardiovascular Medicine, School of Medicine, University of Tokyo, Tokyo, Japan; 6 Department of Preventive Medicine, Northwestern University Feinberg School of Medicine, Chicago, IL 60611 USA

**Keywords:** Anatomy, Cardiology

## Abstract

The prevalence of mitral valve prolapse (MVP) among middle- and older-aged individuals is estimated to be 2–4% in Western countries. However, few studies have been conducted among Asian individuals and young adults. This study included a sample of 2442 consecutive military adults aged 18–39 years in Hualien, Taiwan. MVP was defined as displacement of the anterior or posterior leaflet of the mitral valve to the mid portion of the annular hinge point > 2 mm in the parasternal long-axis view of echocardiography. Cardiac chamber size and wall thickness were measured based on the latest criteria of the American Society of Echocardiography. The clinical features of participants with MVP and those without MVP were compared using a two-sample t test, and the cardiac structures were compared using analysis of covariance with adjustment for body surface area (BSA). Eighty-two participants were diagnosed with MVP, and the prevalence was 3.36% in the overall population. Compared with those without MVP, participants with MVP had a lower body mass index (kg/m^2^) (24.89 ± 3.70 vs. 23.91 ± 3.45, p = 0.02) and higher prevalence of somatic symptoms related to exercise (11.0% vs. 4.9%, p = 0.02) and systolic click in auscultation (18.3% vs. 0.6%, p < 0.01). In addition, participants with MVP had greater left ventricular mass (gm) and smaller right ventricular wall thickness (mm) and dimensions (mm) indexed by BSA than those without MVP (149.12 ± 35.76 vs. 155.38 ± 36.26; 4.66 ± 0.63 vs. 4.40 ± 0.68; 26.57 ± 3.99 vs. 25.41 ± 4.35, respectively, all p-values < 0.01). In conclusion, the prevalence and clinical features of MVP in military young adults in Taiwan were in line with those in Western countries. Whether the novel MVP phenotype found in this study has any pathological meaning needs further investigation.

## Introduction

Mitral valve prolapse (MVP) is one of the most common valvular heart diseases, with a prevalence of 2.0–4.0% in the general population^1–6^. MVP is composed of heterogeneous types with varying clinical presentations. Syndromic MVP commonly coexists with heritable connective tissue disorders such as Ehlers-Danlos syndrome and Marfan syndrome^7–9^, and familial nonsyndromic MVP usually exhibits autosomal dominant inheritance and age- and sex-dependent characteristics^10^. Other types are grouped as sporadic nonsyndromic MVP. A floppy mitral valve plays a pivotal role in the pathogenesis of MVP^11^ and can be assessed by imaging and physical examinations. Systolic click in cardiac auscultation is a typical finding for floppy mitral valve, and the prevalence is estimated from 10 to 70% in patients with MVP^1,12^. A specific heart sound has emerged in patients after 30 years of age, and the intensity increases with more severe MVP and decreases with a higher grade of mitral regurgitation^11,13^. In addition, several somatic symptoms, also called MVP syndrome, such as chest pain, palpitation, and dyspnea, appear early in adolescent and young adult patients, possibly due to low stroke volume and autonomic neural feedback related to MVP^11^.

Although the Framingham Heart Study^1^ has shown that MVP is regarded as a benign condition with a low incidence of complications in the general population, numerous studies have uncovered several adverse events, such as ischemic stroke^14^, bacterial endocarditis^15,16^, severe mitral regurgitation^2,4,17^, lethal arrhythmia^18,19^ and sudden cardiac death^20^ related to MVP. In addition to the Framingham Heart Study that mainly Caucasian participants^21,22^, the clinical characteristics of MVP were also investigated in a cohort of American Indians in the Strong Heart Study^2^, and in a small sample of Canadian adolescents of European, South Asian and Chinese descent in the Study of Health Assessment and Risk in Ethnic groups (SHARE)^5^. Notably, the SHARE study revealed no ethnic differences in the prevalence and clinical features of MVP. However, there has been no large population study carried out in Asia investigating the epidemiology of MVP. Therefore, the purpose of this study was to determine the prevalence, clinical features and echocardiographic profiles of MVP in a large Asian military cohort in Taiwan^23,24^.

## Methods

### Study population

The study used 2442 young male and female adults in the military, aged 18–39 years, from the cardiorespiratory fitness and hospitalization events in armed forces (CHIEF) study performed in Taiwan during 2014–2018^25^. All participants underwent a comprehensive physical examination, and completed a self-reported questionnaire for their family history of inherited diseases and experience regarding toxic substance use, somatic symptoms such as easy palpitation, fatigue, or dyspnea during exercise and physical activity in the Hualien Armed Forces General Hospital of Taiwan. Of these, there were a total of 2442 participants undergoing transthoracic echocardiography for their rank promotions and awards. The rationale and design of the CHIEF study have been described in detail previously^23,24,26,27^.

### Measurements of transthoracic echocardiography

All procedures of transthoracic echocardiography using a 1–5 MHz transducer (iE33; Philips Medical Systems, Andover, MA, USA) were performed by the same experienced and certified technician and verified by the cardiologist (Lin GM) at the Hualien-Armed Forces General Hospital. All participants were examined using parasternal long-axis and short-axis approaches and apical four- and two-chamber views in supine and left lateral positions. According to the suggestions of the American Society of Echocardiography^28^, quantification of LV wall thickness (interventricular septal and posterior walls) and chamber dimension were measured approximately at the onset of the QRS complex of end diastole and tips of the mitral valve by M-mode and two-dimensional measurements in parasternal long axis view. In the parasternal long-axis view, MVP was defined as a displacement of the anterior or posterior leaflet of the mitral valve to the mid portions of the mitral annular hinge point > 2 mm^1,14,29–31^. LV mass was thus calculated according to the corrected formula proposed by Devereux et al.^32^. LV mass = 0.8 × {1.04 × [(left ventricular internal diameter at end diastole (LVIDd) + end diastolic posterior wall thickness + end diastolic interventricular septal thickness]^3^ − LVIDd^3^} + 0.6. Echocardiographic LV hypertrophy was defined as LV mass divided by body surface area (BSA) based on the Dubois formula^33^, as the cutoff value ≥ 88 g/m^2,34^. Measurements of anterior right ventricular (RV) wall thickness were performed by dimensional windows and M-mode at the onset of the QRS complex of end diastole via parasternal long-axis approaches^35^. Echocardiographic RV hypertrophy was defined as anterior RV wall thickness > 5.2 mm, which was the 95th percentile in our young adult cohort in Taiwan^36^.

### Statistical analysis

Characteristics and echocardiographic parameters of the participants with MVP and those without MVP are expressed as the mean ± standard deviation (SD) for continuous variables and numbers (percentage) for categorical variables, respectively. Continuous variables were compared by two-sample t test and categorical variables were compared by the chi-square or Fisher’s exact test. Dimensions of aortic root, left atrial dimension, LV chamber, RV outflow tract, and thickness of LV and RV wall, and LV mass were compared using analysis of covariance (ANCOVA) with adjustment for BSA. A two-tailed value of P < 0.05 was considered significant. All analyses were performed using SAS version 9.4 (SAS Institute Inc, Cary, NC, USA).

### Ethics statement

This study was reviewed and approved by the Institutional Review Board of Mennonite Christian Hospital (No. 16-05-008) in Taiwan, and written informed consent was obtained from all participants. All methods were performed in accordance with the relevant guidelines and regulations.

### Ethics approval and consent to participate

Since there was no research ethics committee regularly held in the Hualien Armed Forces General Hospital, this study applied to the Institutional Review Board (IRB) of the Mennonite Christian Hospital (No. 16-05-008) in Hualien, Taiwan, to approve access to the data for the CHIEF Heart study, and written informed consent was obtained from all participants.

## Results

### Clinical features and laboratory findings

Of the participants in the CHIEF study, there were 2154 males (88.2%) and 288 females (11.8%) participants (Table 1). Eighty-two participants (3.3%) had MVP, which was more prevalent in males (77 of 2154 [3.6%] in males vs. 5 of 288 [1.7%] in females). Participants with MVP had lower levels of body mass index (23.78 kg/m^2^ vs. 24.65 kg/m^2^, P = 0.03) and fasting plasma glucose (90.3 mg/dL vs. 93.63 mg/dL, P = 0.01), and had a higher prevalence of intolerable somatic symptoms including chest pain, dyspnea or easy palpitation during exercise (MVP syndrome, 9 of 82 [11.0%]) than those without MVP. In addition, 7 of the 82 participants with MVP (8.5%) were found to have mild pectus excavatum, a kind of chest wall abnormality, which was a higher percentage than among those without MVP (0.6%, p < 0.01). There were no significant differences in waist circumference, BSA, heart rate, systolic blood pressure, diastolic blood pressure, alcohol consumption, tobacco smoking, serum creatinine, total cholesterol, high-density lipoprotein, low-density lipoprotein, or hemoglobin concentrations. There was a sex difference in diastolic blood pressure. Females with MVP had a lower diastolic blood pressure than those without MVP (54.4 mmHg vs. 64.5 mmHg, P < 0.01), while this difference did not appear in males.

**Table 1:** Characteristics of young male and female adult participants with mitral valve prolapse and those without.

	Total (n = 2442)			Males (n = 254)			Females (n = 288)		
	MVP (−) (n = 2360)	MVP (+) (n = 82)	p-value	MVP (−) (n = 2077)	MVP (+) (n = 77)	p-value	MVP (−) (n = 283)	MVP (+) (n = 5)	p-value
Age	27.36 ± 5.62	26.51 ± 5.82	0.18	27.51 ± 5.70	26.48 ± 5.89	0.12	26.29 ± 4.88	27.00 ± 5.15	0.74
Height (cm)	170.78 ± 6.75	171.58 ± 5.97	0.28	172.12 ± 5.79	172.34 ± 5.19	0.74	160.93 ± 4.90	159.98 ± 5.50	0.66
Weight (kg)	72.12 ± 12.79	70.17 ± 11.68	0.17	73.83 ± 12.24	71.11 ± 11.38	0.05	59.52 ± 9.27	55.66 ± 4.75	0.35
Body mass index (kg/m^2^)	24.65 ± 3.69	23.78 ± 3.43	0.03	24.89 ± 3.70	23.91 ± 3.45	0.02	22.94 ± 3.16	21.81 ± 2.53	0.42
Body surface area (m^2^)	1.84 ± 0.19	1.82 ± 0.17	0.33	1.87 ± 0.17	1.84 ± 0.16	0.09	1.62 ± 0.14	1.57 ± 0.07	0.37
Waist circumference (cm)	82.57 ± 9.99	81.08 ± 10.03	0.18	83.59 ± 9.80	81.51 ± 10.05	0.06	75.10 ± 8.04	74.40 ± 7.47	0.84
Systolic blood pressure (mm Hg)	118.07 ± 13.72	116.50 ± 15.64	0.31	119.65 ± 13.30	117.32 ± 15.43	0.13	106.41 ± 10.90	103.80 ± 14.62	0.59
Diastolic blood pressure (mm Hg)	70.04 ± 10.40	70.03 ± 12.02	0.97	70.78 ± 10.43	71.01 ± 11.35	0.85	64.52 ± 8.31	54.40 ± 12.44	< 0.01
Heart rate (beats/minute)	66.72 ± 10.83	65.44 ± 10.87	0.29	66.53 ± 10.89	65.44 ± 11.09	0.39	68.17 ± 10.26	65.40 ± 7.40	0.54
Somatic symptoms	116 [4.9]	9 [11.0]	0.02	96 [4.6]	7 [9.1]	0.07	20 [7.0]	2 [40.0]	0.06
Pectus excavatum	13 [0.6]	7 [8.5]	< 0.01	12 [0.6]	6 [7.8]	< 0.01	1 [0.4]	1 [20.0]	< 0.01
Current alcohol intake	1048 [44.4]	43 [52.4]	0.15	995 [47.9]	43 [55.8]	0.17	53 [18.7]	0 [0.0]	0.28
Current cigarette smoking	967 [41.0]	30 [36.6]	0.42	912 [43.9]	30 [39.0]	0.39	55 [19.4]	0 [0.0]	0.27
Serum creatinine (mg/dL)	0.92 ± 0.14	0.94 ± 0.13	0.36	0.96 ± 0.12	0.96 ± 0.12	0.98	0.68 ± 0.09	0.66 ± 0.06	0.51
Total cholesterol (mg/dL)	171.00 ± 33.36	167.43 ± 29.08	0.33	171.62 ± 33.87	167.56 ± 29.76	0.29	166.40 ± 29.02	165.40 ± 17.01	0.93
HDL-C (mg/dL)	49.78 ± 10.69	50.50 ± 10.60	0.54	48.57 ± 9.97	50.12 ± 10.80	0.18	58.67 ± 11.61	56.40 ± 3.78	0.66
LDL-C (mg/dL)	103.85 ± 28.93	99.71 ± 25.83	0.20	105.49 ± 29.23	100.13 ± 26.34	0.11	91.80 ± 23.33	93.20 ± 16.56	0.89
Serum triglyceride (mg/dL)	104.01 ± 84.10	91.16 ± 56.49	0.17	108.95 ± 87.48	91.86 ± 57.46	0.08	67.69 ± 36.24	80.40 ± 41.72	0.43
Fasting plasma glucose (mg/dL)	93.63 ± 11.89	90.30 ± 9.76	0.01	94.01 ± 12.34	90.25 ± 9.77	< 0.01	90.85 ± 7.22	91.20 ± 10.52	0.91
Hemoglobin (g/dL)	14.97 ± 1.25	15.12 ± 1.24	0.28	15.25 ± 0.96	15.28 ± 0.80	0.82	12.87 ± 1.10	12.62 ± 1.11	0.61

Continuous variables are expressed as mean ± SD (standard deviation), and categorical variables as N [%].

*HDL-C* high-density lipoprotein cholesterol, *LDL-C* low-density lipoprotein cholesterol, *MVP* mitral valve prolapse.

### Echocardiographic findings in all participants

**Figure 1:**
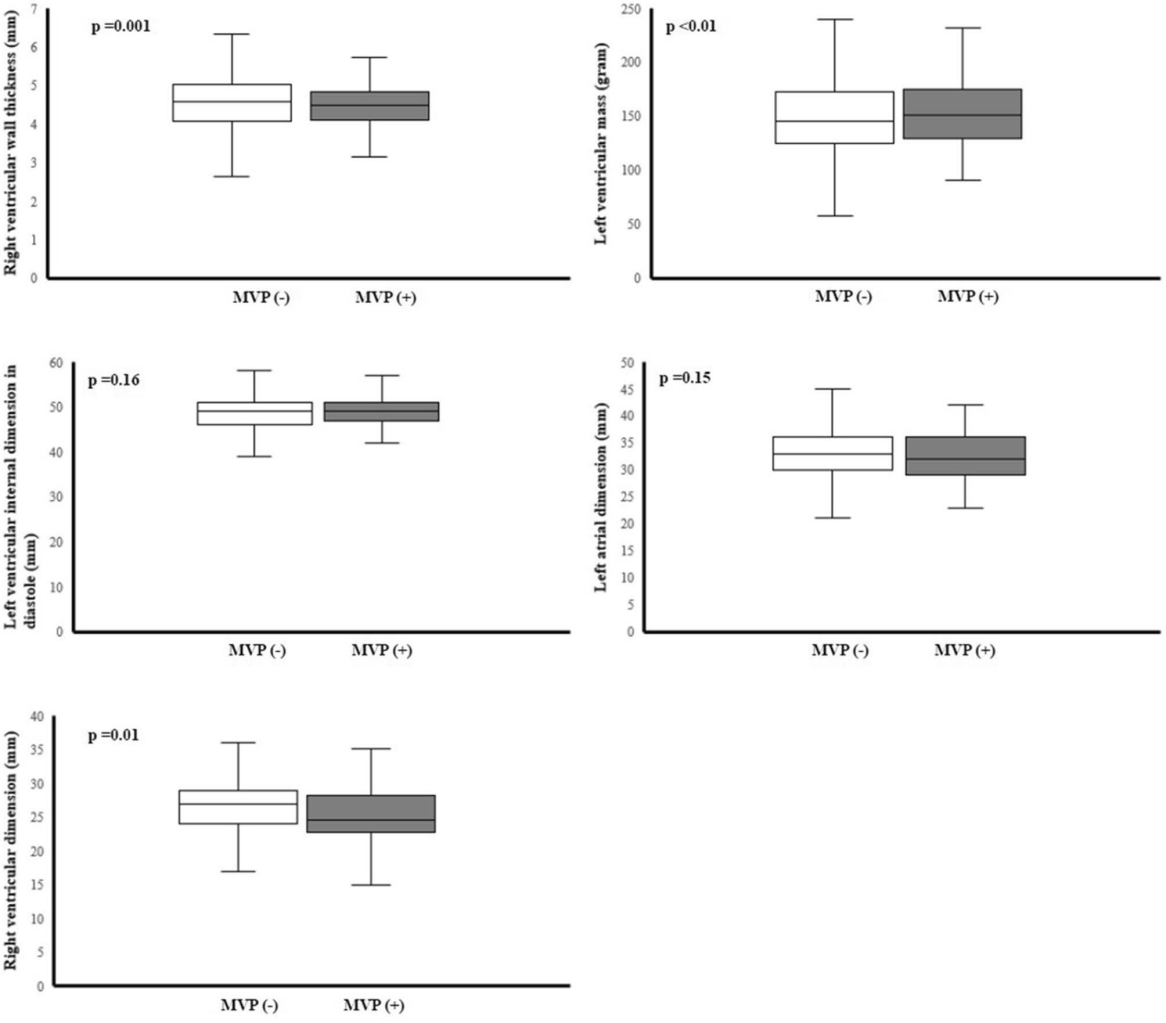
Echocardiographic left atrial dimension, left ventricular internal dimension in diastole, left ventricular mass, right ventricular outflow tract dimension in diastole and right ventricular wall thickness in the overall population were compared using analysis of covariance with adjustment for body surface area.

**Figure 2:**
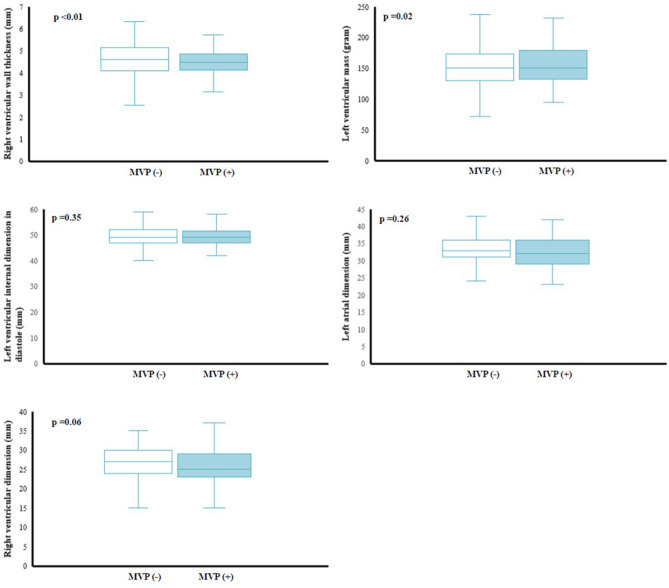
Echocardiographic left atrial dimension, left ventricular internal dimension in diastole, left ventricular mass, right ventricular outflow tract dimension in diastole and right ventricular wall thickness in males were compared using analysis of covariance with adjustment for body surface area.

**Figure 3:**
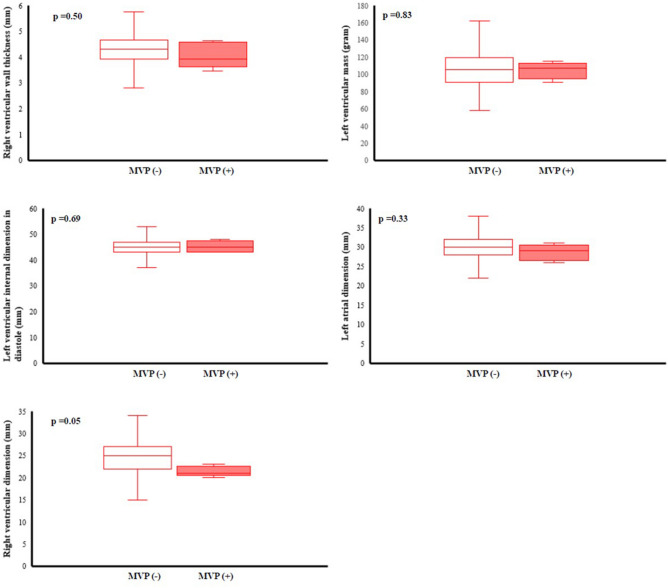
Echocardiographic left atrial dimension, left ventricular internal dimension in diastole, left ventricular mass, right ventricular outflow tract dimension in diastole and right ventricular wall thickness in females were compared using analysis of covariance with adjustment for body surface area.

Table 2 showed the results of a comparison of the cardiac geometry and function assessment between groups. With regard to the cardiac geometry adjusted for BSA, there were no differences in the aortic root, LV chamber, or the left atrial dimension between groups. However, the participants with MVP had lower levels of RV outflow tract dimension in diastole (25.41 ± 4.35 mm vs. 26.57 ± 3.99 mm, P = 0.01) and RV wall thickness (4.40 ± 0.68 mm vs. 4.66 ± 0.63 mm, *P* = 0.001) than those without MVP. In contrast, there were greater levels of LV mass (155.38 ± 36.26 gm vs. 149.12 ± 35.76 gm, P < 0.01), interventricular septal thickness (8.95 ± 1.13 mm vs. 8.74 ± 1.21 mm, P = 0.02) and LV posterior wall thickness (8.73 ± 1.13 mm vs. 8.53 ± 1.11 mm, P = 0.01) in the participants with MVP than those without MVP. It is also notable that the pattern of RV geometry adjusted for BSA in MVP in the overall participants (Fig. 1) were consistent in both males (Fig. 2) and females (Fig. 3), whereas the pattern of a greater LV mass in MVP was only presented in males but not females. Participants with MVP had a higher prevalence of mitral regurgitation, at least mild grade, than those without MVP (84.1% vs. 74.6%, *P* = 0.04), but showed no differences in the prevalence of aortic, pulmonary or tricuspid regurgitation or tricuspid valve prolapse. In addition, there was no difference in LV ejection fraction between groups. With regard to the diastolic filling, the participants with MVP had a higher E/A ratio (1.95 ± 0.46 vs. 1.81 ± 0.50, *P* = 0.01) and a lower late diastolic peak A velocity (45.87 ± 9.83 cm/s vs. 49.46 ± 10.72 cm/s, *P* < 0.01) but no difference in early diastolic peak E velocity.

**Table 2:** Echocardiographic findings of young male and female participants with mitral valve prolapse and those without.

	Total (n = 242)			Males (n = 254)			Females (n = 288)		
	MVP (−) (n = 2360)	MVP (+) (n = 82)	p-value	MVP (−) (n = 2077)	MVP (+) (n = 77)	p-value	MVP (−) (n = 283)	MVP (+) (n = 5)	p-value
Cardiac geometry*									
Aortic valve open (mm), PLAX	19.84 ± 2.04	19.46 ± 2.08	0.15	20.13 ± 1.91	19.68 ± 1.96	0.10	17.75 ± 1.67	16.20 ± 0.45	0.05
Aortic root dimension (mm), PALX	29.53 ± 3.53	29.48 ± 2.67	0.87	29.95 ± 3.44	29.74 ± 2.53	0.78	26.40 ± 2.34	25.40 ± 1.14	0.55
LV mass (gm), PLAX	149.12 ± 35.76	155.38 ± 36.26	< 0.01	154.82 ± 33.55	158.70 ± 34.82	0.02	107.26 ± 20.44	104.21 ± 9.73	0.83
LV posterior wall (mm), PLAX	8.53 ± 1.11	8.73 ± 1.13	0.01	8.69 ± 1.05	8.84 ± 1.08	0.03	7.36 ± 0.79	7.00 ± 0.00	0.47
LV internal dimension in diastole (mm), PLAX	48.78 ± 3.71	49.10 ± 3.40	0.16	49.26 ± 3.51	49.35 ± 3.32	0.35	45.22 ± 3.16	45.20 ± 2.28	0.69
LV internal dimension in systole (mm), PLAX	30.56 ± 3.44	30.64 ± 3.39	0.74	30.94 ± 3.38	30.73 ± 3.45	0.93	27.87 ± 2.55	29.00 ± 1.72	0.37
Interventricular septum, (mm), PLAX	8.74 ± 1.21	8.95 ± 1.13	0.02	8.91 ± 1.15	9.04 ± 1.11	0.08	7.48 ± 0.85	7.60 ± 0.55	0.45
RV wall thickness (mm)^a^, PLAX	4.66 ± 0.63	4.40 ± 0.68	< 0.01	4.70 ± 0.62	4.42 ± 0.69	< 0.01	4.33 ± 0.58	4.07 ± 0.50	0.50
RV outflow tract dimension in diastole (mm), PLAX	26.57 ± 3.99	25.41 ± 4.35	0.01	26.79 ± 3.97	25.68 ± 4.35	0.06	24.96 ± 3.76	21.40 ± 1.14	0.05
Left atrial dimension (mm), PLAX	32.88 ± 4.06	32.10 ± 4.17	0.15	33.22 ± 4.06	32.32 ± 4.18	0.26	30.38 ± 3.03	28.60 ± 2.07	0.33
LV hypertrophy	114 [4.8]	3 [3.7]	0.62	102 [4.9]	3 [3.9]	0.68	12 [4.2]	0 [00.0]	0.63
RV hypertrophy^a^	206 [10.1]	2 [2.9]	0.04	187 [10.5]	2 [3.1]	0.05	19 [7.5]	0 [00.0]	0.52
**Cardiac functional assessment**									
LV ejection fraction (%), PLAX	61.90 ± 5.06	62.61 ± 4.23	0.21	61.97 ± 5.13	62.64 ± 4.32	0.26	61.43 ± 4.44	62.20 ± 2.59	0.70
Tricuspid valve prolapse, PSAX	388 [16.4]	17 [20.7]	0.30	350 [16.9]	16 [20.8]	0.36	38 [13.4]	1 [20.0]	0.67
Aortic regurgitation ≥ mild grade	38 [1.6]	1 [1.2]	0.78	34 [1.6]	1 [1.3]	0.81	4 [1.4]	0 [0.0]	0.78
Mitral regurgitation ≥ mild grade	1760 [74.6]	69 [84.1]	0.04	1544 [74.3]	65 [84.4]	0.04	216 [76.3]	4 [80.0]	0.84
Pulmonary regurgitation ≥ mild grade	1442 [61.1]	53 [64.6]	0.51	1293 [62.3]	50 [64.9]	0.63	149 [52.7]	3 [60.0]	0.74
Tricuspid regurgitation ≥ mild grade	1884 [79.8]	62 [75.6]	0.35	1657 [79.8]	60 [77.9]	0.69	227 [80.2]	2 [40.0]	0.02
RV systolic pressure (mmHg)	27.31 ± 4.67	26.63 ± 4.62	0.19	27.39 ± 4.70	26.81 ± 4.66	0.29	26.73 ± 4.35	23.80 ± 2.78	0.13
Peak E velocity (cm/s)	85.84 ± 15.49	86.26 ± 14.70	0.81	84.69 ± 15.02	86.02 ± 15.03	0.44	93.91 ± 17.17	89.90 ± 8.41	0.60
Peak A velocity (cm/s)	49.46 ± 10.72	45.87 ± 9.83	< 0.01	49.28 ± 10.67	46.01 ± 10.06	< 0.01	50.80 ± 11.04	43.78 ± 5.41	0.15
E/A ratio	1.81 ± 0.50	1.95 ± 0.46	0.01	1.79 ± 0.49	1.94 ± 0.48	0.01	1.94 ± 0.57	2.06 ± 0.10	0.65
Peak E′ velocity (cm/s)	16.73 ± 5.72	15.98 ± 3.19	0.24	16.56 ± 5.93	15.94 ± 3.23	0.37	17.99 ± 3.70	16.54 ± 2.80	0.38
Peal A′ velocity (cm/s)	8.30 ± 3.17	7.34 ± 1.98	< 0.01	8.28 ± 3.27	7.34 ± 2.01	0.01	8.47 ± 2.27	7.31 ± 1.48	0.25
E′/A′ ratio	2.19 ± 1.08	2.34 ± 0.84	0.21	2.18 ± 1.12	2.34 ± 0.85	0.21	2.26 ± 0.69	2.35 ± 0.64	0.76

Continuous variables are expressed as mean ± SD (standard deviation), and categorical variables as N [%].

*BSA* body surface area, *LV* left ventricle, *RV* right ventricle, *PLAX* echocardiographic parasternal long axis view, *PSAX* echocardiographic parasternal short axis view

^a^N = 2102 in total (N = 1842 for males and N = 260 for females).

*indicates the p-value for the echocardiographic geometry variables with adjustment for body surface area.

### Echocardiographic findings in participants without mitral regurgitation

Since the LV and RV geometry could be influenced by the presence of mitral regurgitation, the echocardiographic characteristics in those free of mitral regurgitation were also analyzed (n = 613) in Table 3. The results showed that the participants with MVP had similar levels of LV mass, LV chamber size and anterior RV wall thickness indexed by BSA compared to those without MVP. Participants with MVP had a smaller RV outflow tract dimension indexed by BSA (25.23 ± 5.21 mm vs. 27.16 ± 4.04 mm, P = 0.05) and a lower late diastolic peak A velocity (50.80 ± 11.38 cm/s vs. 43.19 ± 9.11 cm/s, *P* = 0.01) than those without MVP.

**Table 3:** Comaprisons of cardiac structure between participants with mitral valve prolapse and those without in absence of mitral regurgitation.

	Participants without mitral regurgitation (n = 613)		
	MVP (−) (n = 600)	MVP (+) (n = 13)	p-value
Cardiac geometry*			
Aortic valve open (mm), PLAX	19.61 ± 2.11	19.54 ± 1.76	0.89
Aortic root dimension (mm), PALX	28.95 ± 4.18	29.85 ± 3.36	0.38
LV mass (gm), PLAX	153.43 ± 34.79	144.73 ± 40.72	0.26
LV posterior wall (mm), PLAX	8.70 ± 1.12	8.53 ± 1.33	0.55
LV internal dimension in diastole (mm), PLAX	48.83 ± 3.66	47.38 ± 2.95	0.11
LV internal dimension in systole (mm), PLAX	30.55 ± 3.72	28.75 ± 2.55	0.13
Interventricular septum, (mm), PLAX	8.94 ± 1.19	8.92 ± 1.65	0.96
RV wall thickness (mm)^a^, PLAX	4.57 ± 0.86	4.42 ± 0.75	0.52
RV outflow tract dimension in diastole (mm), PLAX	27.16 ± 4.04	25.23 ± 5.21	0.05
Left atrial dimension (mm), PLAX	33.11 ± 4.00	32.23 ± 4.69	0.34
LV hypertrophy	46 [7.7]	0 [0.0]	0.29
RV hypertrophy^a^	53 [10.6]	0 [0.0]	0.21
**Cardiac functional assessment**			
LV ejection fraction (%), PLAX	61.31 ± 4.86	62.85 ± 4.86	0.25
Tricuspid valve prolapse, PSAX	70 [11.7]	2 [15.4]	0.68
Aortic regurgitation ≥ mild grade	1 [0.2]	0 [0.0]	0.88
Pulmonary regurgitation ≥ mild grade	263 [43.8]	6 [46.2]	0.86
Tricuspid regurgitation ≥ mild grade	334 [55.7]	5 [38.5]	0.21
RV systolic pressure (mmHg)	26.18 ± 4.44	24.87 ± 3.81	0.29
Peak E velocity (cm/s)	85.42 ± 14.91	82.96 ± 14.73	0.55
Peak A velocity (cm/s)	50.80 ± 11.38	43.19 ± 9.11	0.01
E/A ratio	1.76 ± 0.51	1.96 ± 0.34	0.16
Peak E′ velocity (cm/s)	15.86 ± 3.38	15.04 ± 2.72	0.38
Peal A′ velocity (cm/s)	8.00 ± 2.24	7.28 ± 2.06	0.25
E′/A′ ratio	2.13 ± 0.74	2.26 ± 0.94	0.55

Continuous variables are expressed as mean ± SD (standard deviation), and categorical variables as N [%].

*BSA* body surface area, *LV* left ventricle, *RV* right ventricle, *PLAX* echocardiographic parasternal long axis view, *PSAX* echocardiographic parasternal short axis view

^a^N = 512.

*indicates the p-value for the echocardiographic geometry variables with adjustment for body surface area.

## Discussion

The principal findings in this population-based study were that the prevalence of MVP in an unselected young-aged military cohort in Taiwan was 3.36%, and the clinical features of those with MVP included a higher prevalence of somatic symptoms related to exercise and manifested with both lower body mass index and fasting plasma glucose concentration. In addition, a new phenotype of MVP in cardiac geometry presented by greater LV mass and smaller RV wall thickness and RV dimension adjusted to BSA was observed in military individuals in Taiwan.

This was the second study following a Japanese population study^37^ to provide information on the prevalence and correlates of MVP in a large population in Asia, specifically in Taiwan. The prevalence of MVP reported in a previous case–control study in Taiwan was estimated to be 1.92% in a population of ethnic Taiwanese individuals with a mean age of 49.2 years^36^. However, their study subjects were retrieved from the Nationwide Health Insurance Database in Taiwan, and the cases were selected merely on the basis of the International Classification of Diseases, 9th Revision, Clinical Modification code for MVP^38^, which might underestimate the prevalence of MVP. In the present study, 3.3% of young adults had MVP, falling toward the higher end of the range among previous population-based studies^1,2,5,6,21^. This might be attributed in part to using a displacement over 2 mm of mitral valve as the only criterion for defining MVP, regardless of considering the thickness of leaflets^1,29^ and possibly increasing the sensitivity in detection. In the Coronary Artery Risk Development in Young Adults (CARDIA) study, the prevalence of MVP was estimated to be 0.6% using the same echocardiographic criterion in a similar age group^3^ and is lower than that in our study population. While the CARDIA study comprised biethnic samples of black and white individuals^3^, the ethnic group in this study was Asian adults. Therefore, the discrepancy may arise from the different ethnic groups.

This was consistent with previous study findings that those with MVP presented prevalent somatic symptoms associated with exercise and favorable metabolic features, including leaner body mass index and lower fasting plasma glucose^1,2,5^. Most importantly, the present study showed new associations, independent of BSA, between MVP and biventricular geometry, including greater LV mass and reduced anterior RV wall thickness and dimension. Previous studies on the cardiac geometry of MVP have mainly focused on the left ventricle, and the results have been inconsistent^2,3^. While the CARDIA study showed no difference in LV mass indexed by BSA between the echocardiographic MVP group and the no MVP group in young adults^3^, Devereux et al. displayed lower LV mass indexed by BSA in those with MVP^2^ LV mass growth might be an unfavorable index for cardiovascular disease in MVP^19,39,40^. It is likely that greater LV mass in this cohort is related to underlying myopathy with focal basal inferolateral hypertrophy in the setting of annular curling or, when present, to significant volume load from mitral regurgitation. The mechanism was supported by the finding that those with MVP had similar levels of LV mass compared with those without MVP in the absence of mitral regurgitation in this study. Furthermore, the finding of a lower peak A wave velocity, possibly indicating a status of early left atrial systolic dysfunction in MVP, was consistent between those with mitral regurgitation and those without. With regard to the smaller RV wall thickness and dimension in MVP, the mechanisms are not clear. Whether the specific RV geometry of MVP originates in an inherited manner or is compensated by a lower stroke volume related to MVP needs further investigation^11,41^.

Another interesting finding in this study is that females with MVP presented favorable ventricular geometry, while males with MVP had greater LV mass indexed by BSA and smaller RV wall thickness and dimension. Females with MVP were unlikely to have more mitral regurgitation than males. This anatomical difference in MVP by sex was consistent with the study conducted by Avierinos et al., who documented that more females harbored benign forms of MVP in anatomy (more anterior and bileaflet prolapse, more thickened mitral leaflets, fewer flail leaflets) and physiology (less mitral regurgitation)^42^. However, inconsistent with a previous report^43^, the prevalence of MVP in females was lower than that in males in this study, possibly because of the small number of females (N = 288) included in the present study.

### Study limitations

Our study had some limitations. First, 88% (72/82) of the MVP morphology was presented by elongations of the anterior mitral leaflet (32/82), posterior mitral leaflet (22/82) or both leaflets (18/82) without significant leaflet thickening, and the other 10% (10/82) had focal leaflet thickening, probably due to myxomatous degeneration. Moreover, there were no cases of chordae tendineae ruptures or coexisting congenital heart diseases, such as bicuspid aortic valves. It is possible that young people with familial syndromic MVP were initially excluded at enlistment, leaving most of the cases in the military as familial or sporadic nonsyndromic MVP. Second, our participants were recruited from among military personnel who might not be completely representative of the general population regarding their physically active behavior, which could result in bias such as a greater LV mass in MVP.

## Conclusion

The prevalence and clinical features of MVP in military young adults in Taiwan were in line with those in Western countries. Those with MVP in Taiwan were found to have smaller levels of RV chamber size and RV wall thickness, left atrial systolic dysfunction, and a greater LV mass index. Whether there were any pathological meanings or sequelae for the novel phenotype of MVP found in this study needs further investigation.

## Data Availability

Since the study materials were obtained from the military in Taiwan, the data are confidential and not allowed to be opened in public. If there are any questions for clarification, the readers can contact Dr. Gen-Min Lin, the corresponding author, for sharing the data.
